# Early tumour size changes from neoadjuvant chemotherapy as a predictor of pathologic response in breast cancer

**DOI:** 10.1371/journal.pone.0346704

**Published:** 2026-05-27

**Authors:** Erika Z. Chung, David Alberico, Lakshmanan Sannachi, Archya Dasgupta, Joyce Yip, Maria Lourdes Anzola Pena, Sonal Gandhi, Belinda Curpen, Gregory J. Czarnota

**Affiliations:** 1 Physical Sciences, Sunnybrook Research Institute, Toronto, Canada; 2 Department of Radiation Oncology, Sunnybrook Health Sciences Centre, Toronto, Canada; 3 Department of Radiation Oncology, University of Toronto, Toronto, Canada; 4 Division of Medical Oncology, Department of Medicine, Sunnybrook Health Sciences Centre, Toronto, Canada; 5 Department of Medicine, University of Toronto, Toronto, Canada; 6 Department of Medical Imaging, Sunnybrook Health Sciences Centre, Toronto, Canada; 7 Department of Medical Imaging, University of Toronto, Toronto, Canada; 8 Department of Medical Biophysics, University of Toronto, Toronto, Canada; University of Pisa, ITALY

## Abstract

Neoadjuvant chemotherapy (NAC) is the standard of care for locally advanced, human epidermal growth factor receptor 2-positive, and triple negative breast cancer. While complete pathologic response is associated with improved outcomes, NAC response varies and is only assessed post-surgery. Identifying response earlier can allow for timely treatment personalization, reducing toxicity and costs as well as improving efficacy. This study aimed to assess the feasibility of using early changes in tumour size, as measured by ultrasound during chemotherapy at 6 times, to predict final pathological response to NAC in breast cancer patients. Eligible participants were ≥18 years with biopsy-proven stage l-lll breast cancer who received standard NAC and underwent ultrasound imaging at weeks 0, 1, 4, 8, 12, and 16 during chemotherapy. A modified response grading system based on post-operative histopathological evaluation was used to classify patients as Complete Responders (CR), Partial Responders (PR), Weak Responders (WR), or Non-Responders (NR). Changes in tumour diameter, area, and volume were evaluated as early predictors of final pathologic response. Statistical analysis was performed to assess changes from baseline to each time point across pairs of groups. A total of 106 patients were included. Post-NAC response rates were assessed for each group. CRs displayed steep reductions in diameter, area, and volume in the early weeks of NAC, while PRs showed more consistent and moderate decreases. WRs demonstrated less pronounced reductions, and NRs remained largely unchanged across all time points. Statistical analysis confirmed differences in tumour size changes between CRs and other groups (p < 0.05), supporting the observed trends in diameter, area, and volume reductions. Overall, early changes in tumour size may provide some predictive value for final pathologic response to NAC but are likely insufficient on their own, underscoring the need for more robust predictive markers.

## Introduction

While breast cancer mortality has decreased by 42% since its peak in 1989, incidence rates have been steadily increasing by about 0.5% a year since the mid-2000s due in part to improved detection and a greater prevalence of reproductive and lifestyle risk factors [1,2]. Today, breast cancer is not only the most commonly diagnosed cancer in women, accounting for nearly a third of all new cancer diagnoses, but it is also the leading cause of cancer death in women worldwide [1,2].

Locally advanced breast cancer (LABC), which generally includes inoperable stage lllB and lllC tumours, as well as those with human epidermal growth factor receptor 2 (HER2) positive and triple negative breast cancer (TNBC) carry an elevated risk of recurrence and poorer overall survival [3–7]. That said, achieving local control can offer the potential for a cure [3]. As a result, neoadjuvant chemotherapy (NAC) administered with the goal of downstaging tumour to improve operability, followed by reassessment for surgery, is the standard of care for these patients [3].

Nevertheless, treatment response is incredibly heterogeneous in oncology, and it typically takes multiple treatment cycles to identify poor or non-responders of therapy. Accordingly, decisions to adapt treatment are typically deferred until after chemotherapy is completed, by which time months have been spent on futile treatment and resources only while the cancer progresses. Currently, there are limited methods capable of reliably distinguishing responders from non-responders early in the treatment course. Prognostic assessment and subsequent treatment planning remains by and large dependent on molecular profiling and pathological factors such as TNM staging [3,8]. Thus, a method for predicting pathologic response would be invaluable for enabling timely, personalized treatment plans that maximize therapeutic benefit and avoid unnecessary toxicity and costs, ultimately helping to improve patient outcomes.

Building on prior work that explored early treatment response in breast cancer patients using an ultrasound-based radiomics approach, this study investigates an alternative potential marker of treatment response. The present study aims to characterize early tumor size changes assessed by ultrasound during NAC associated with pathologic response, with the goal of assessing their feasibility for guiding treatment decisions and supporting personalized care in medicine.

## Materials and methods

### Study population

This study included patients enrolled from June 1, 2018 to September 30, 2023 who were over the age of 18 with biopsy-proven clinical stage l-lll breast cancer, as defined by the American Joint Committee on Cancer (AJCC) 7th edition, and treated with NAC [9]. Patients with axillary nodes seen on staging scans or on palpation underwent an ultrasound-guided biopsy for pathologic confirmation. NAC regimens included: AC-T (adriamycin, cyclophosphamide, paclitaxel), AC-TH (AC-T plus trastuzumab), FEC-D (5-fluorouracil, epirubicin, cyclophosphamide, docetaxel), FEC-DH (FEC-D plus trastuzumab) and KEYNOTE 522 (pembrolizumab, paclitaxel, carboplatin then pembrolizumab, doxorubicin, cyclophosphamide).

Patients were required to have normal blood counts, creatinine levels, liver function tests, and cardiac function. Exclusion criteria included a history of connective tissue or dermatologic diseases involving the breast, or an Eastern Cooperative Oncology Group (ECOG) performance status ≥3. This study received approval from the institutional ethics committee (Sunnybrook Research Institute; project ID 185–2006, superseded by SUN-1994 in 2020 when they migrated to a new computer system) and was registered on clinicaltrials.gov (NCT00437879) on February 20, 2007, where the protocol is available (https://clinicaltrials.gov/study/NCT00437879). All participants provided written informed consent, and the study adhered to the Declaration of Helsinki.

Imaging Acquisition and Segmentation Ultrasound imaging for each patient was performed at week 0, 1, 4, 8, 12, 16 of NAC by experienced sonographers. We have previously shown that the inter-observer variation in ultrasound measurements are minimal [10]. A Sonix RP clinical ultrasound system (Analogic Medical Corp., Massachusetts, USA) with a L14-5W/60 linear array transducer (centre frequency 6.5 MHz, bandwidth range 3–8 MHz) was used to image the primary tumour volume at evenly spaced 5 mm intervals across the tumour mass. At each time point, the image frame displaying the greatest dimensions was selected. One image frame was obtained from a pan view and another from a perpendicular reference view, which provided perspectives in all three spatial plans allowing for three-dimensional analysis. Region of interests (ROIs), i.e., the primary tumour, were manually contoured in MATLAB R2022b (Mathworks Inc, Natick, MA).

### Tumour response definition

Magnetic resonance imaging (MRI) was performed as part of standard clinical care prior to the initiation of chemotherapy to evaluate tumour extent and serve as a reference for baseline tumour measurement for clinical use. Following completion of neoadjuvant treatment, response was subsequently assessed based on residual tumour size and cellularity on the resected histopathology specimen. Patients were categorized as responders (R) or nonresponders (NR) using a modified response grading system based on histopathological evaluation [11].

The responder category included patients who demonstrated a reduction in the diameter of tumour by at least 30% from baseline imaging to the surgical specimen histopathology, or a decrease in cellularity to less than 5% in the tumour bed (partial responders, PR), or the complete disappearance of all target lesions (further subclassified as complete responders, CR). In cases (infrequent) where there was a diminishment in cellularity to less than 5% but not a diminishment in >30% of tumour size, they were subclassified as Weak Responders (WR). The non-response (NR) category included patients with a tumour size reduction of less than 30% and cellularity greater than 5%.

### Tumour size analysis

Four metrics of tumour size were determined from each patient’s contoured pan and reference ROIs using a custom MATLAB application. The metrics included geometric area, diameter, an axial measurement, and a lateral measurement. The geometric area represented the area of the polygon-shaped ROI (mm^2^). The diameter was defined as the longest distance between any two boundary points on the ROI (mm). The axial measurement was described as the longest distance between any two boundary points parallel to the axial axis (mm), while the lateral measurement represented the longest distance between any two points parallel to the lateral axis (mm). Volume was another metric of interest. Tumour volume was estimated by assuming an ellipsoid shape and applying the formula *V = 1/6πabc*, where *a* was calculated as the average of the axial pan and reference measurements, *b* the lateral pan measurement, and *c* the lateral reference measurement.

To compare changes from baseline across groups at each week a Mann-Whitney test for two independent samples was performed using Real Statistics in Excel 2016. The null hypothesis was that there was no observable difference in the mean changes in volume size from baseline to week x between a pair of groups in breast cancer patients. The alternative hypothesis was that there was an observable difference in the mean changes in volume size from baseline to week x between a pair of groups in breast cancer patients. Two-tailed *P* values less than 0.05 were considered significant.

## Results

The study cohort was comprised of 106 breast cancer patients. A summary of patient and treatment characteristics is provided in Table 1. The average age of patients was 50 (–IQR 42–57). The median pre-treatment tumor size along the longest axis, i.e., diameter, as measured by MRI for baseline purposes was 4.0 cm (IQR 3.1–6.1). The majority had grades ll-lll tumours (96%). The most common molecular subtype was estrogen receptor (ER)-positive, progesterone receptor (PR)-positive, and HER2-negative. The most common NAC regimen was dose-dense AC-T (±H) (63%), followed by FEC-D (±H) 26(%) and KEYNOTE 522 (6%). Based on histopathological evaluation after NAC and surgery, the rates of CR, PR, WR, and NR were 20%, 67%, 8%, 5%, respectively. A per patient breakdown pf patient and treatment characteristics is provided in S1 Table. Compliance with the ultrasound at baseline, weeks 1, 4, 8, 12 and 16 were 100%, 91%, 95%, 70%, 54% and 46%, respectively.

**Table 1:** Patient and treatment characteristics.

Characteristic	n = 106
Median age, years (IQR)	50 (–42 - 57)
Median initial tumour size by MRI, cm (IQR)	4.0 (3.1–6.1)
Side, n (%) Left Right Bilateral	46 (43%) 59 (56%) 1 (1%)
Grade, n (%) l ll lll	4 (4%) 50 (47%) 52 (49%)
Molecular Markers, n (%) ER/PR + HER2- ER/PR + HER2+ ER/PR- HER2+ ER/PR/HER2-	46 (43%) 21 (20%) 16 (15%) 23 (22%)
Histological Type, n (%) IDC ILC Other	98 (92%) 3 (3%) 5 (5%)
Chemotherapy, n (%) AC-T AC-TH FEC-D FEC-DH KEYNOTE 522 Other	52 (49%) 15 (14%) 19 (18%) 9 (8%) 6 (6%) 5 (5%)
Treatment Response, n (%) Complete Responder Partial Responder Weak Responder Non-Responder	21 (20%) 71 (67%) 9 (8%) 5 (5%)

Abbreviations: interquartile range, IQR; estrogen receptor, ER; progresterone receptor, PR; human epidermal growth factor receptor 2, HER2; chemotherapy regimens: AC-T (adriamycin, cyclophosphamide, paclitaxel), AC-TH (AC-T plus trastuzumab), FEC-D (5-fluorouracil, epirubicin, cyclophosphamide, docetaxel), FEC-DH (FEC-D plus trastuzumab), KEYNOTE 522 (pembrolizumab, paclitaxel, carboplatin then pembrolizumab, doxorubicin, cyclophosphamide)

**Fig 1:**
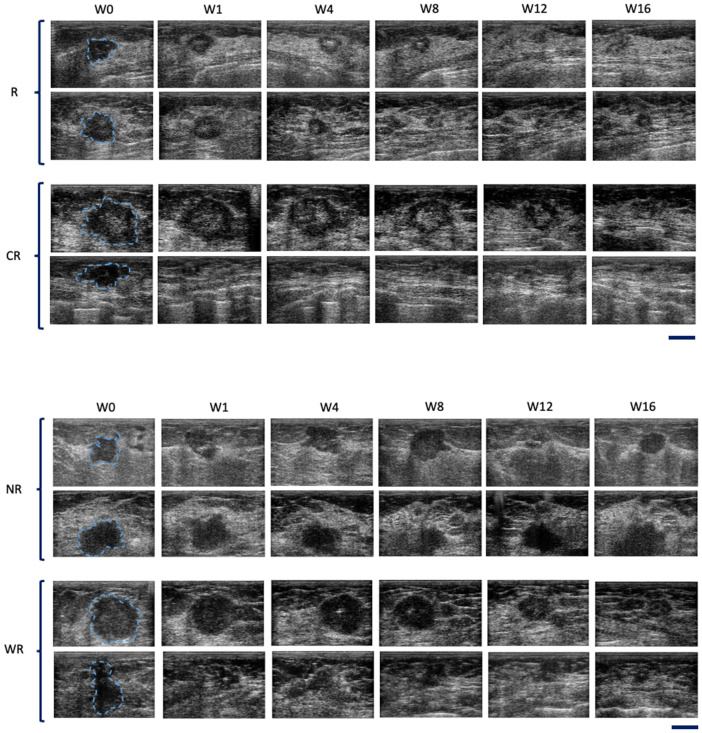
B-mode imaging at weeks 0, 1, 4, 8, 12, and 16 for two representative patients from CR, PR, WR, and NR groups. The scale bar represents a length of 1 cm.

**Fig 2:**
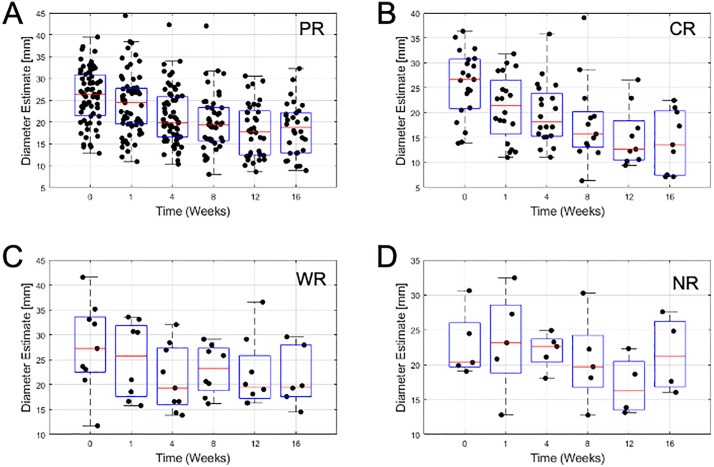
Tumour diameter estimates. **A)** Partial Responders **B)** Complete Responders **C)** Weak Responders **D)** Non-Responders. Box plots show boxes from lower quartile to upper quartile. The red line indicates the median value. Whiskers indicate minimum and maximum values. Outliers may lie outside whiskers.

**Fig 3:**
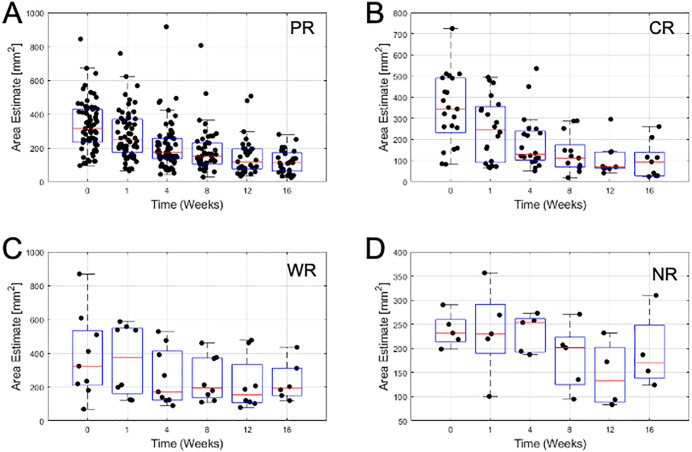
Tumour area estimates. **A)** Partial Responders **B)** Complete Responders **C)** Weak Responders **D)** Non-Responders. Box plots show boxes from lower quartile to upper quartile. The red line indicates the median value. Whiskers indicate minimum and maximum values. Outliers may lie outside whiskers.

**Fig 4:**
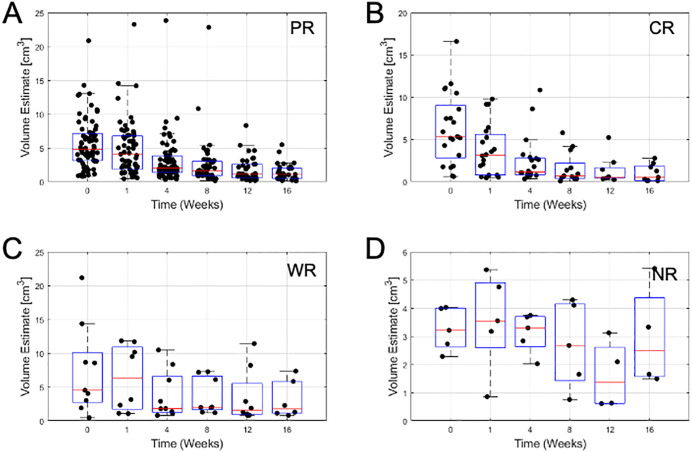
Tumour volume estimates. **A)** Partial Responders **B)** Complete Responders **C)** Weak Responders **D)** Non-Responders. Box plots show boxes from lower quartile to upper quartile. The red line indicates the median value. Whiskers indicate minimum and maximum values. Outliers may lie outside whiskers.

**Fig 5:**
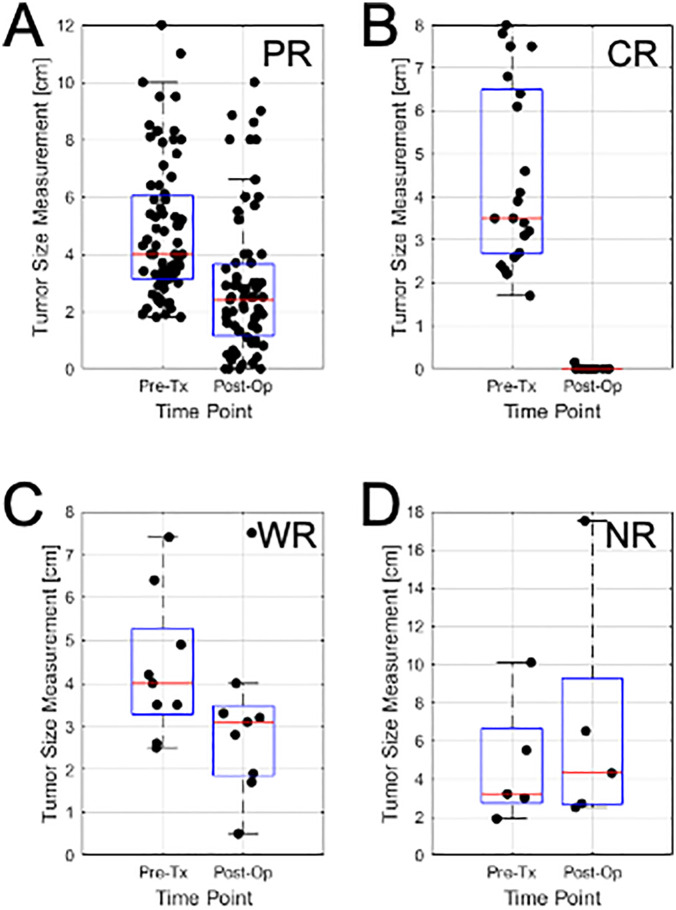
Tumor size estimates from pre-treatment (MRI) and post-operative (surgical report) measurements. **A)** Partial Responders **B)** Complete Responders **C)** Weak Responders **D)** Non-Responders.. Box plots show boxes from lower quartile to upper quartile. The red line indicates the median value. Whiskers indicate minimum and maximum values. Outliers may lie outside whiskers.

From ultrasound B-mode images (Fig 1) a size diminishment in Complete responders and partial responders was obvious within 1–8 weeks. Weak responders (which had a size diminishment less than 30% in the longest dimension) appeared to respond less rapidly during chemotherapy. Non-responders had disease that was invariant in size throughout chemotherapy. Responding patients had some increase in backscatter that was obvious but not quantified here, In Fig 2 data presented demonstrates the size changes for all four response groups at measured times for tumour diameter. Figs 3,4 similarly show data for tumour area and volume, respectively. Differences were obvious between all four groups with CR patients exhibiting the greatest and quickest change in tumour sizes. Fig 5 indicates changes in all groups before and after treatment.

**Fig 6:**
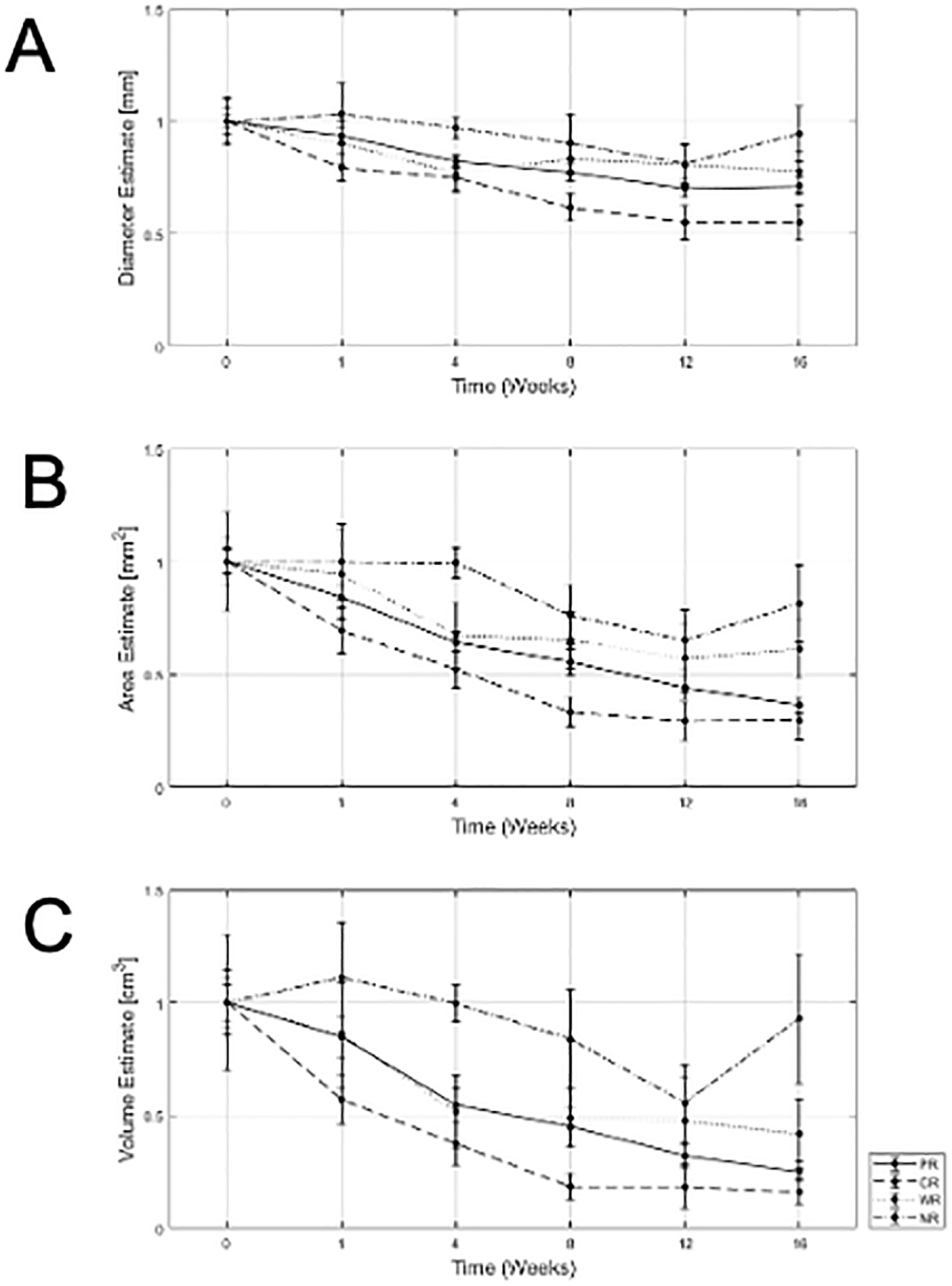
Tumor size changes with time. **A)** Partial Responders **B)** Complete Responders **C)** Weak Responders **D)** Non-Responders. Plots show mean and standard error of the mean.

Table 2 presents the mean sizes of tumours using different measures at different times. Relative size changes are presented in Fig 2. Partial responders diminished in size slower than complete responders; at week 1 partial responders had diminished to 84 ± 42% whereas complete responders had further diminished to 70 ± 44% of their initial size. Weak responders exhibited a minor change to 95 ± 55% whereas non-responders show a 100 ± 37% size. By week 16 partial responders had diminished to 36 ± 20% and complete responders had diminished to 29 ± 24% of their original size in terms of cross-sectional area. Weak responders and non-responders were at 61 ± 31% and 81 ± 34% of their original sizes. Changes in diameter and volume followed similar trends (Fig 6).

**Table 2:** Size measurements for tumour groups at different times (mean ± SD).

Time	Week 0	Week 1	Week 4	Week 8	Week 12	Week 16
**Diameter** (mm)						
**PR**	25.9 ± 6.3	24.2 ± 7.1	21.3 ± 6.2	20.0 ± 6.4	18.2 ± 6.1	18.5 ± 6.0
**CR**	25.9 ± 6.5	20.6 ± 6.7	19.3 ± 6.3	16.0 ± 5.6	14.2 ± 6.2	14.2 ± 6.2
**WR**	27.6 ± 9.0	25.0 ± 7.7	21.2 ± 6.6	23.0 ± 5.0	22.2 ± 7.1	21.4 ± 6.0
**NR**	22.7 ± 5.0	23.5 ± 7.0	22.1 ± 2.4	20.5 ± 6.5	18.4 ± 3.8	21.5 ± 5.6
**Area** (mm^2^)						
**PR**	329 ± 14	277 ± 140	211 ± 134	183 ± 130	145 ± 107	119 ± 67
**CR**	343 ± 164	238 ± 153	179 ± 127	114 ± 78	101 ± 83	101 ± 85
**WR**	379 ± 248	359 ± 211	256 ± 168	247 ± 134	218 ± 161	233 ± 118
**NR**	237 ± 34	237 ± 88	236 ± 36	180 ± 70	155 ± 63	194 ± 81
**Volume**(mm^4^)						
**PR**	5608 ± 3735	4755 ± 4015	3064 ± 3352	2519 ± 3512	1797 ± 1769	1412 ± 1254
**CR**	6288 ± 4170	3587 ± 3136	2360 ± 3737	1164 ± 1323	1149 ± 1684	1016 ± 1010
**WR**	7400 ± 6688	6343 ± 4852	3824 ± 3563	3611 ± 2708	3526 ± 4026	3103 ± 2781
**NR**	3192 ± 780	3547 ± 1772	3180 ± 590	2670 ± 1569	1770 ± 1086	2967 ± 1824

Across all three measurements made (tumour volume, tumour cross-sectional area, and tumour diameter), comparisons were made between response groups with respect to size changes between baseline (week 0) and each subsequent time point (weeks 1, 4, 8, 12 and 16). P-values for each statistical test are provided in Tables 3–5. In general, comparisons of tumour size data between CR and PR patients frequently demonstrated significant differences at W1 (p < 0.05), suggesting more rapid initial shrinkage in CR than in PR. By contrast, measurement from tumours in CR versus WR patients showed no significant differences when assessed by volume and diameter, but differences became evident when tumour area was considered. CR versus NR generally demonstrated significant differences at all time points, with the notable exception of W12 when assessed by volume and area. WR versus R showed overlapping trajectories in size change when analyzed by volume and diameter, with both indicating no significant differences at all weeks. However, comparisons of area revealed significant differences from W4 onward. WR versus NR showed inconsistent patterns across volume, diameter, and area. NR versus PR showed significant differences after W1 when assessed by volume and area, but changes in diameter did not follow this trend. No one size measurement (diameter, area or volume) appeared to be better than another.

**Table 3:** Mann-Whitney Test for Two Independent Samples for Diameter.

	W0-W1	W0-W4	W0-W8	W0-W12	W0-W16
**CR v PR**	0.016*	0.233	0.198	0.246	0.072
**CR v WR**	0.508	0.945	0.246	0.064	0.560
**CR v NR**	0.017*	0.063	0.026	0.041*	0.009
**WR v PR**	0.548	0.396	0.833	0.357	0.435
**WR v NR**	0.143	0.027*	0.305	0.395	0.008*
**NR v PR**	0.085	0.068	0.072	0.078	0.019*

**Table 4:** Mann-Whitney Test for Two Independent Samples for Area.

	W0-W1	W0-W4	W0-W8	W0-W12	W0-W16
**CR v PR**	0.181	0.346	0.378	0.552	0.062
**CR v WR**	0.309	0.002*	<0.001*	0.092	0.001*
**CR v NR**	0.020*	0.010*	0.020*	0.089	0.005*
**WR v PR**	0.251	<0.001*	, < 0.001*	0.028*	<0.001*
**WR v NR**	0.464	0.0196	0.003	0.174	0.010
**NR v PR**	0.093	0.011*	0.055	0.028*	0.004*

**Table 5:** Mann-Whitney Test for Two Independent Samples for Volume.

	W0-W1	W0-W4	W0-W8	W0-W12	W0-W16
**CR v PR**	0.018*	0.230	0.428	0.613	0.151
**CR v WR**	0.309	0.511	0.425	0.462	0.723
**CR v NR**	0.006*	0.008*	0.016	0.089	0.005*
**WR v PR**	1	0.974	0.797	0.476	0.269
**WR v NR**	0.464	0.060	0.078	0.496	0.019*
**NR v PR**	0.084	0.0219*	0.026*	0.038*	0.003*

## Discussion

NAC is typically administered in a fixed regimen over several months, with final overall treatment response most often determined through histopathological evaluation after completion of chemotherapy and surgery. Consequently, some patients discover that treatment was ineffective only after undergoing a full course of chemotherapy, exposing them to unnecessary toxicity and delayed initiation of alternative potentially more efficacious therapies. Methods capable of predicting treatment responses earlier in the treatment course are invaluable for enabling personalized NAC. For example, predicted responders may undergo dose de-escalation to reduce toxicity to organs at risk (OARs), while predicted non-responders may be switched earlier to palliative schedules or alternative regimens.

Pathologic response was chosen as the primary outcome of the current study, as complete pathological response (pCR) remains the best surrogate for long-term outcomes of local control and disease-free survival in patients with breast cancer [12]. Indeed, the I-SPY2 adaptively randomized clinical trial reported that patients with pCR (vs non-pCR) had better 3-year event-free survival (95% vs 78%, p < 0.001) and distant recurrence-free survival (95% vs 81%, p < 0.001) [13]. These outcomes were observed irrespective of treatment regimen, which in all cases consisted of a taxane-based therapy with or without one of the several investigational agents or combinations followed by doxorubicin and cyclophosphamide [13]. The association between pCR and superior outcomes was also supported by Kim et al., who reported that patients with HER2-overexpressing breast cancer who achieved pCR after NAC with concurrent trastuzumab had higher 5-year rates of locoregional recurrence-free (100% versus 95%, *P* = 0.011), distant metastasis-free (96% versus 80%, *P* < 0.001), recurrence-free (96% versus 79%, *P* < 0.001), and overall survival (95% versus 84%, *P* = 0.006) [14].

To the best of current knowledge, this study is the first to evaluate early size changes by ultrasound across multiple time points during NAC as potential predictors of pathologic response in breast cancer. Previous work by Adrada et al. examined size changes from ultrasound images during NAC, but their analysis was limited to a single time point (after 2cycles of NAC) and to patients with TNBC [15]. Moreover, their study focused solely on tumour volume, whereas the present study assesses several methods of tumour size estimation, including volume, area, and diameter [15]. Notably, Adrada et al. demonstrated that early volumetric changes may have prognostic value, as patients who achieved a tumour volume reduction of ≥80% after two cycles of NAC had a pCR rate of 77% compared to 29% for those with a percent reduction <80% [15].

Other studies have explored different approaches to using size changes to predict NAC outcomes. For example, Wang et al. identified ultrasound-based cut-off values for diameter reduction that correlated with different grades of pathologic response in patients with invasive breast cancer and integrated these with other clinicopathological variables and ultrasound parameters to construct a prognostic nomogram [16]. Likewise, Cui et al. developed a reasonably reliable two-cycle response nomogram based on ultrasound and clinicopathological features, highlighting tumour shrinkage as a key determinant of pCR [17]. A separate prognostic model combined circulatory tumour cell changes, tumour size changes, and stromal tumour-infiltrating lymphocyte levels to achieve an AUC of 0.945 for predicting pCR across molecular subtypes [18]. By contrast, another study found that the value of mid-treatment ultrasound measurements for predicting residual cancer burden may vary by molecular subtype [19]. Recently, we found that the change in tumour size after 4 weeks of neoadjuvant chemotherapy versus baseline was not as predictive as quantitative ultrasound radiomics changes at the same time point. However, after longer time intervals (i.e., completion of neoadjuvant chemotherapy), change in tumour size change was more significantly different than baseline [11].

Tumour response to NAC is currently determined using magnetic resonance imaging (MRI) and positron emission tomography (PET) scans taken before chemotherapy and upon completion of chemotherapy pre-operatively. These imaging methods have been investigated for early response prediction for NAC. For example, Cao et al. explored early ultrafast dynamic contrast-enhanced (DCE) MRI parameters over four time points during NAC, reporting area under the curves for predicting pCR ranging from 0.70 to 0.81 [20]. Additionally, Kawamura et al. identified two MRI patterns of early response to chemotherapy for mass-type breast cancer: shrinkage of the contrasted area on DCE MRI and an increase in the apparent diffusion coefficient [21]. Furthermore, the multicenter ACRIN 6698 trial demonstrated that changes in tumour apparent diffusion coefficient at diffusion-weighted MRI after 12 weeks of NAC can predict pathologic response, with pCR patients showing greater increases in ADC from baseline levels compared to patients non-pCR patients [22].

In contrast, the AVATAXHER randomized phase 2 trial performed fluorodeoxyglucose (FDG) PET scans before the second cycle of NAC in patients with early HER2-positive breast cancer [23]. PET-predicted non-responders were subsequently randomized to either continue standard docetaxel and trastuzumab therapy or receive intensified therapy with the addition of bevacizumab [23]. The latter was found to lead to a higher pathological complete response rate (44% versus 24%), which demonstrates the clinical utility of early treatment adaptation [23]. Additional PET-based strategies have further underscored the prognostic value of early tumor changes to assess response to NAC. In the TNBC subtype, early reductions in tumour blood flow measured by FDG PET after the first cycle of NAC strongly predicted outcomes, with 3- year overall survival observed in 100% of women with both pCR and blood flow response versus 48% of women with no pCR and no blood flow response [24]. In inflammatory breast cancer, a ≥ 72% reduction in tumor glucose metabolism (SUVmax) assessed by 18F-FDG PET/CT between baseline and pre-surgery independently predicted distant metastasis-free survival [25]. Similarly, in ER-rich postmenopausal breast cancer, baseline SUVmax of 18F-FDG PET/CT identified patients more likely to benefit from NAC than neoadjuvant endocrine therapy, with 18F-FES-negative tumours associated with significantly higher rates of disease-free survival and lower rates of recurrence when treated with NAC [26].

In the present study, ultrasound was explored as a monitoring modality due to its practicality. Not only is ultrasound more widely available than PET and MRI, but it is also lower-cost, portable, relatively safe, and fast, making it well-suited for integration into routine clinical care [27]. Importantly, this study demonstrated that early changes in tumor size, assessed across multiple time points with ultrasound during NAC, show potential as predictive markers of treatment response.

Although early tumour size changes have not been widely examined for predictive value, radiomics has emerged as a promising strategy for early prognostic assessment in recent years. Radiomics involves extracting quantitative features that capture underlying microstructural tissue characteristics normally imperceptible to the human eye from standard imaging modalities (US, CT, MRI, and PET) using advanced machine learning techniques. Recent work, including the companion study to this paper by our group, demonstrated the feasibility of ultrasound-based radiomics in predicting tumour shrinkage according to RECIST criteria in breast cancer patients. In that investigation, a previously developed quantitative ultrasound (QUS) classification model based on a support vector machine-radial basis function (SVM-RBF) algorithm trained on QUS and texture features from over 200 breast tumours in an independent cohort achieved high performance metrics (92% accuracy, 83% sensitivity, 93% specificity, and 99% positive predictive value) [28].

This study has several limitations. First, the number of non-responders was small, limiting the generalizability of findings. Future studies should validate these findings in larger cohorts, especially those with a greater number of non-responders, and across multiple centres. Second, tumour volume calculations assumed an ellipsoid shape. While this approximation may not reflect true tumour geometry, it was considered acceptable given the study’s focus on relative volume changes rather than absolute changes. Nonetheless, more advanced volumetric methods could potentially provide greater accuracy in future studies. Third, we acknowledge that multiple pairwise comparisons is not ideal, but all comparisons were pre-planned. Fourth, our study included 106 patients, which is insufficient to perform subgroup analyses of different histologies and subtypes. Finally, some imaging data were unavailable due to missed appointments patient compliance. Future works include running separate analyses by molecular subtypes and chemotherapy regimens to determine whether early size changes differ across groups and comparing size changes with quantitative ultrasound features to explore potential associations, but this will require larger sample sizes.

## Conclusion

In summary, the work here suggests that early changes in tumour size may provide some value in monitoring pathologic response to NAC in breast cancer patients. However, early tumour size changes are likely insufficient on their own. These findings underscore the need for more robust predictive markers to support early identification of non-responders and guide timely treatment adaptation for improved patient outcomes.

## Supporting information

S1 Table Individual patients’ tumour and treatment characteristics, and pathologic response. (DOCX)
