# Supplementary material for: Early tumour size changes from neoadjuvant chemotherapy as a predictor of pathologic response in breast cancer
Source: PLoS One. 2026 May 27;21(5):e0346704. doi: 10.1371/journal.pone.0346704 (PMC13215535; doi:10.1371/journal.pone.0346704)
Supplement: S1 Table — (DOCX) [file pone.0346704.s001.docx]

| **Patient #** | **Side** | **Size** | **Histologic Type** | **Grade** | **ER** | **PR** | **HER** | **Chemotherapy** | **Response** |
| --- | --- | --- | --- | --- | --- | --- | --- | --- | --- |
| 1 | Left | 1.9 | IDC | 3 | - | - | - | TC | R |
| 2 | Right | 1.9 | IDC | 3 | - | - | - | AC-T | NR |
| 3 | Right | 3.6 | IDC | 3 | - | - | + | AC-T/H | R |
| 4 | Right | 3.3 | IDC | 3 | + | + | - | AC-T | R |
| 5 | Left | 4.8 | IDC | 3 | + | + | - | AC-T | R |
| 6 | Right | 3.5 | IDC | 3 | + | + | - | AC-T | NR |
| 7 | Right | 3.5 | IDC | 3 | + | + | - | AC-T | NR |
| 8 | Left | 4.4 | IDC | 2-3 | + | + | - | FEC-D | R |
| 9 | Right | 6.7 | IDC | 3 | + | + | - | AC-T | R |
| 10 | Right | 2.2 | IDC | 3 | + | - | - | AC-T | R |
| 11 | Right | 8.5 | IDC | 3 | + | - | - | AC-T | R |
| 12 | Right | 6.1 | IDC | 1-2 | + | + | - | FEC-D | R |
| 13 | Right | 8 | IDC | 2 | + | + | + | AC-T/H4 | R |
| 14 | Right | 2.3 | IDC | 2 | - | - | + | FEC-D/H2 | CR |
| 15 | Right | 3.4 | IDC | 2 | + | + | - | AC-T | R |
| 16 | Left | 4 | IDC | 3 | + | + | - | FEC-D | R |
| 17 | Right | 3.4 | IDC | 2 | + | + | - | FEC-D | CR |
| 18 | Right | 3.5 | IDC | 3 | + | + | + | FEC-D/H3 | CR |
| 19 | Left | 4 | IDC | 3 | - | - | - | AC-T | R |
| 20 | Right | 3.5 | IDC | 3 | + | - | - | TC | WR |
| 21 | Right | 3 | IDC | 1 | + | + | - | AC-T | R |
| 22 | Right | 6.4 | IDC | 2 | - | - | + | FEC-D | R |
| 23 | Right | 7.5 | IDC | 2-3 | + | - | - | AC-T | CR |
| 24 | Left | 3.1 | IDC | 3 | - | - | - | AC-T | CR |
| 25 | Right | 7.4 | IDC | 2 | + | + | - | FEC-D | WR |
| 26 | Left | 7.3 | IDC | 1-2 | - | - | - | FEC-D/H3 | R |
| 27 | Left | 1.7 | IDC | 3 | + | - | + | AC-T/H | CR |
| 28 | Left | 5.6 | IDC | 2-3 | - | - | - | AC-T | R |
| 29 | Right | 10 | IDC | 3 | - | - | - | AC-T | R |
| 30 | Left | 12 | ILC | 1-2 | + | + | - | FEC-D | R |
| 31 | Left | 1.8 | IDC | 2-3 | - | - | + | AC-T | R |
| 32 | Right | 2.6 | IDC | 2 | + | + | - | FEC-D | R |
| 33 | Right | 2.4 | IDC | 2 | + | - | - | AC-T | R |
| 34 | Right | 3.2 | IDC | 3 | - | - | - | AC-T | R |
| 35 | Right | 3.4 | IDC | 1 | + | + | - | AC-T | R |
| 36 | Right | 9.5 | IDC - MICROPAPILLARY | 3 | + | + | - | AC-T | R |
| 37 | Right | 2.4 | IDC | 3 | - | - | - | AC-T | R |
| 38 | Left | 2.5 | IDC | 3 | - | - | + | AC-T/H4 | R |
| 39 | Left | 3.6 | IDC | 2 | - | - | - | TC | R |
| 40 | Left | 3.5 | IDC | 2 | + | - | + | FEC-D | CR |
| 41 | Left | 3.5 | IDC | 3 | + | - | + | FEC-D/H3 | R |
| 42 | Left | 3.6 | IDC | 3 | + | + | - | AC-T | R |
| 43 | Left | 11 | IDC | 2 | + | + | - | FEC-D | R |
| 44 | Left | 2.9 | IDC | 3 | - | - | + | FEC-D | R |
| 45 | Left | 6.4 | IDC | 3 | + | + | - | AC-T | CR |
| 46 | Left | 8.3 | IDC | 3 | + | + | - | AC-T | R |
| 47 | Left | 2.3 | IDC | 2 | - | - | - | AC-T | R |
| 48 | Right | 2.4 | IDC | 3 | - | - | - | AC-T | CR |
| 49 | Left | 7.5 | IDC | 2 | + | + | - | AC-T | R |
| 50 | Right | 2.9 | IDC | 2 | + | + | - | AC-T | R |
| 51 | Right | 2.6 | IDC | 2 | + | + | - | FEC-D | WR |
| 52 | Right | 3.2 | IDC | 2 | + | + | - | FEC-D | R |
| 53 | Left | 5.2 | IDC | 2 | + | + | - | FEC-D | R |
| 54 | Right | 3.5 | IDC | 2 | + | + | - | FEC-D | R |
| 55 | Right | 9.5 | IDC | 2 | + | + | - | FEC-D | R |
| 56 | Left | 4 | IDC | 2 | - | - | + | FEC-D/H3 | R |
| 57 | Right | 6.8 | IDC | 2 | - | - | + | FEC-D/H4 | CR |
| 58 | Right | 10.1 | IDC | 2 | - | - | - | AC-T | NR |
| 59 | Right | 3.3 | IDC | 3 | + | + | + | AC-T | R |
| 60 | Right | 2.1 | IDC | 2 | + | + | - | AC-T | R |
| 61 | Left | 4 | ILC | 3 | + | + | - | AC-T | R |
| 62 | Left | 5.3 | IDC | 2 | + | - | + | FEC-D/H3 | R |
| 63 | Left | 4.9 | INVASIVE APOCRINE | 2 | - | - | - | AC-T | R |
| 64 | Left | 5.3 | IDC and lobular | 2-3 | + | - | - | AC-T | R |
| 65 | Left | 2.8 | IDC | 3 | + | - | - | AC-T | R |
| 66 | Right | 7.8 | Invasive micropapillary | 2 | - | - | + | AC-T/H4 | CR |
| 67 | Left | 7.1 | IDC | 2 | - | - | + | AC-T/H4 | R |
| 68 | Right | 4.6 | IDC | 2 | - | - | - | AC-T | CR |
| 69 | Left | 8 | MUCINOUS | 2 | + | + | - | AC-T | R |
| 70 | Right | 4.5 | IDC | 2 | + | + | - | AC-T | R |
| 71 | Right | 3.1 | IDC | 3 | - | - | - | AC-T | R |
| 72 | Right | 1.8 | IDC | 2-3 | + | - | + | FEC-D/H3 | R |
| 73 | Left | 4.3 | IDC | 2 | - | - | + | AC-T/H4 | R |
| 74 | Left | 7.5 | IDC WITH EXTENSIVE NECROSIS | 3 | - | - | - | AC-T | CR |
| 75 | Right | 2.5 | IDC - MICROPAPILLARY | 1-2 | + | + | - | AC-T | WR |
| 76 | Right | 1.9 | IDC | 2 | + | + | + | AC-T/H4 | R |
| 77 | Left | 5 | IDC | 2 | + | - | + | FEC-D/H3 | R |
| 78 | Right | 8.1 | MICROPAPILLARY | 3 | - | - | + | AC-T/H4 | R |
| 79 | Left | 4 | IDC | 2 | + | + | + | AC-T/H4 | WR |
| 80 | Left | 3.9 | ILC | 2-3 | + | - | + | AC-T/H4 | CR |
| 81 | Right | 2.3 | IDC | 2 | + | + | + | AC-T/H4 | R |
| 82 | Right | 4.9 | DUCTAL/mucinous | 3 | + | + | - | AC-T | WR |
| 83 | Right | 5 | IDC | 2 | + | + | - | AC-T | R |
| 84 | Right | 5.4 | IDC | 3 | - | - | + | AC-T/H4 | R |
| 85 | Right | 5.9 | METAPLASIC | 3 | + | + | - | AC-T | R |
| 86 | Right | 3.7 | IDC | 2 | - | - | - | KEYNOTE-522 | R |
| 87 | Left | 3.8 | INFLAMATORY | 3 | - | - | - | AC-T | R |
| 88 | Right | 7.9 | IDC | 2 | + | - | - | FEC-D | R |
| 89 | Left | 4 | MICROPAPILLARY | 1-2 | + | + | - | AC-T | R |
| 90 | Left | 4.1 | IDC | 3 | - | - | + | AC-T/ H4 | CR |
| 91 | Left | 6.4 | IDC/EXTENSIVE DCIS | 2-3 | + | + | - | AC-T | WR |
| 92 | Right | 3.2 | IDC | 2 | + | - | - | KEYNOTE-522 | CR |
| 93 | Left | 3.4 | IDC | 2 | + | + | + | DOCE/carbo | R |
| 94 | Right | 2.7 | IDC | 3 | - | - | - | KEYNOTE-522 | CR |
| 95 | Right | 4.2 | IDC | 2 | + | + | - | AC-T | WR |
| 96 | Bilateral | 5.9 | IDC | 1 | + | - | - | AC-T | R |
| 97 | Right | 6.4 | IDC | 2 | - | - | + | AC-T/H4 | R |
| 98 | Left | 4.3 | IDC | 3 | + | + | - | AC-T | R |
| 99 | Right | 5.5 | DUCTAL/Lobular | 3 | + | + | - | FEC-D | NR |
| 100 | Left | 2.6 | IDC | 2 | - | - | - | KEYNOTE-522 | CR |
| 101 | Right | 2.1 | Invasive Micropapillary | 1 | + | + | - | FEC-D | R |
| 102 | Left | 2.2 | IDC | 2-3 | - | - | - | AC-T | CR |
| 103 | Right | 3.5 | IDC | 2 | + | + | - | DOCE/CARBO | WR |
| 104 | Left | 6.1 | IDC | 2 | + | + | + | AC-T | CR |
| 105 | Right | 5.4 | IDC | 3 | - | - | + | KEYNOTE-522 | R |
| 106 | Left | 8.0 | IDC | 3 | - | - | - | KEYNOTE-522 | CR |

Abbreviations: IDC, invasive ductal carcinoma; ILC, invasive lobular carcinoma; ER, estrogen receptor; PR, progesterone receptor; CR, complete response; R, partial response; WR, weak response; NR, no response; chemotherapy regimens: AC-T (adriamycin, cyclophosphamide, paclitaxel), AC-TH (AC-T plus trastuzumab), FEC-D (5-fluorouracil, epirubicin, cyclophosphamide, docetaxel), FEC-DH (FEC-D plus trastuzumab), KEYNOTE 522 (pembrolizumab, paclitaxel, carboplatin then pembrolizumab, doxorubicin, cyclophosphamide).
